# EEG Complexity Analysis of Psychogenic Non-Epileptic and Epileptic Seizures Using Entropy and Machine Learning

**DOI:** 10.3390/e27101044

**Published:** 2025-10-07

**Authors:** Hesam Shokouh Alaei, Samaneh Kouchaki, Mahinda Yogarajah, Daniel Abasolo

**Affiliations:** 1 Centre for Biomedical Engineering, School of Engineering, University of Surrey, Guildford GU2 7XH, UK; d.abasolo@surrey.ac.uk; 2 Centre for Vision, Speech and Signal Processing (CVSSP), University of Surrey, Guildford GU2 7XH, UK; samaneh.kouchaki@surrey.ac.uk; 3 Department of Clinical and Experimental Epilepsy, Institute of Neurology, University College London, London WC1N 3BG, UK; m.yogarajah@ucl.ac.uk

**Keywords:** psychogenic non-epileptic seizures, epileptic seizures, entropy, preictal and interictal analysis, machine learning

## Abstract

Psychogenic non-epileptic seizures (PNES) are often misdiagnosed as epileptic seizures (ES), leading to inappropriate treatment and delayed psychological care. To address this challenge, we analysed electroencephalogram (EEG) data from 74 patients (46 PNES, 28 ES) using one-minute preictal and interictal recordings per subject. Nine entropy measures (Sample, Fuzzy, Permutation, Dispersion, Conditional, Phase, Spectral, Rényi, and Wavelet entropy) were evaluated individually to classify PNES from ES using k-nearest neighbours, Naïve Bayes, linear discriminant analysis, logistic regression, support vector machine, random forest, multilayer perceptron, and XGBoost within a leave-one-subject-out cross-validation framework. In addition, a dynamic state, defined as the entropy difference between interictal and preictal periods, was examined. Sample, Fuzzy, Conditional, and Dispersion entropy were higher in PNES than in ES during interictal recordings (not significant), but significantly lower in the preictal (*p* < 0.05) and dynamic states (*p* < 0.01). Spatial mapping and permutation-based importance analyses highlighted O1, O2, T5, F7, and Pz as key discriminative channels. Classification performance peaked in the dynamic state, with Fuzzy entropy and support vector machine achieving the best results (balanced accuracy = 72.4%, F1 score = 77.8%, sensitivity = 74.5%, specificity = 70.4%). These results demonstrate the potential of entropy features for differentiating PNES from ES.

## 1. Introduction

Psychogenic non-epileptic seizures (PNES) present a considerable clinical challenge due to their close resemblance to epileptic seizures (ES) in terms of clinical manifestations such as convulsions and altered behaviour [1]. However, the underlying mechanisms differ significantly: while ES are the result of abnormal, synchronous electrical discharges in the brain, PNES are manifestations of psychological distress and are not associated with electrophysiological abnormalities during events [2]. This symptomatic overlap contributes to a high rate of misdiagnosis. It is estimated that 10–20% of patients referred to epilepsy centres with presumed refractory epilepsy are eventually diagnosed with PNES [3]. Misdiagnosis may result in unnecessary administration of anti-epileptic drugs, exposure to their potential side effects, increased healthcare costs, and delays in accessing appropriate psychological interventions [4].

Currently, video-electroencephalography (video-EEG) is considered the gold standard for distinguishing PNES from ES [5]. Nevertheless, there are several obstacles to video-EEG monitoring, e.g., not all epileptic convulsions exhibit observable ictal abnormalities in EEG signals [6]. Moreover, video-EEG is costly, time-consuming, inconvenient for patients, and not available in all hospitals because it requires inpatient hospitalisation and prolonged recording [5].

Entropy analysis has emerged as a promising approach for capturing subtle distinctions in brain dynamics. Entropy measures quantify non-linear and irregular fluctuations in physiological signals and are sensitive to changes in neural complexity [6,7]. Given their ability to characterise signal irregularity, entropy methods have shown promise in differentiating PNES from ES by revealing subtle complexity changes not captured by conventional EEG analyses [6,8].

Early ictal studies attempted to discriminate PNES from ES during seizure episodes. In a small cohort (4 ES, 7 PNES), Pippa et al. extracted Shannon entropy (ShEn) and logarithmic-energy entropy (LogEn) alongside conventional time/frequency features and applied ReliefF feature selection [9]. Although Naïve Bayes (NB) achieved 95% accuracy, entropy features were not among the top-ranked predictors. Building on this, Ahmadi et al. studied a larger EEG ictal data from 20 PNES and 20 ES using ShEn, spectral entropy (SpecEn), and Rényi entropy (RenEn) combined with fractal dimensions, including Higuchi fractal dimension (HFD) and Katz fractal dimension (KFD) [8]. After feature selection with the Imperialist Competitive Algorithm, they compared multiple classifiers and reported 95.03% accuracy, with SpecEn and RenEn emerging as the most informative features [8]. Complementary evidence of the effectiveness of SpecEn was provided by Vega-Zelaya et al., who reported a significant reduction during bursts of periodic activity (BPA) compared to ES when ictal epochs were normalised to pre- and post-burst intervals [10]. This finding illustrates SpecEn’s sensitivity to rhythmic or periodic EEG structure.

Given the confounding effects of muscle artefacts in ictal EEG, later studies shifted attention to non-ictal data. Hinchliffe et al. (2022) evaluated a broader range of entropy algorithms—including Approximate entropy (ApEn), Sample entropy (SampEn), SpecEn, RenEn, singular value decomposition Entropy (SVDEn), and Wavelet entropy (WaveEn)—on preictal and interictal EEGs [6]. The best result was obtained with k-Nearest Neighbours (kNN) using RenEn (~95% accuracy). However, this result was based on epoch-wise ten-fold cross-validation, in which samples from the same subject appeared in both training and testing sets, thereby inflating the reported accuracy [6]. Other interictal studies have confirmed the utility of SampEn. In one investigation, Sample Entropy (SampEn) achieved a higher area under the receiver operating characteristic curve (AUC = 0.76) than delta power (AUC = 0.5) for distinguishing non-epileptic episodes (NEE) from temporal lobe epilepsy (TLE) using logistic regression (LR) [11]. Lu et al. combined SampEn with HFD to distinguish epileptic from non-epileptic EEG segments, achieving 89.8% accuracy using a support vector machine (SVM) and reporting significantly lower entropy in epileptic signals [12]. However, the study focused on differences within EEG epochs rather than between patient groups.

The potential of other entropy metrics has also been explored. Permutation Entropy (PermEn) did not differ significantly between PNES and healthy controls (HC) in preictal EEG; nonetheless, it proved valuable for interpreting DL models [13]. PermEn was used within stacked autoencoders (SAE) and convolutional neural networks (CNNs), where entropy in feature maps decreased progressively with layer depth, highlighting its role as an interpretability marker [13,14]. Collectively, these studies demonstrate that SampEn, SpecEn, and RenEn are promising for PNES–ES classification, while PermEn provides insight into model behaviour, particularly in deep architectures. Table 1 summarises the results of prior work that applied entropy algorithms to EEG analysis for PNES-ES classification.

Despite these encouraging findings, several limitations constrain the clinical translation of this body of work. Cohorts are often small and heterogeneous, with comparator groups including HC, BPA, or specific epilepsy subtypes (e.g., TLE). Validation strategies based on epoch-level splits for diagnostic group classification are prone to data leakage and overestimate performance. Another unresolved issue is the exploration of alternative entropy techniques. Fuzzy entropy (FuzzyEn), for instance, has been shown to outperform ShEn, RenEn, ApEn, and SampEn in distinguishing epileptic from non-epileptic EEG within the patients with ES [15], yet has not been applied to EEG signals from PNES groups. Moreover, most analyses relied on global or averaged entropy values, neglecting the possibility that specific regions may be more informative for PNES–ES differentiation. Only limited evidence exists on the spatial distribution of entropy changes in the brain. For example, Allendorfer et al. applied SampEn to resting-state fMRI in traumatic brain injury (TBI), reporting reduced entropy in the frontoparietal network, and observed that SampEn was higher in both PNES and ES patients with prior TBI than in TBI-only participants [16]. Using both EEGs and electrocardiograms (ECGs), a study revealed significant amplitude differences in heartbeat-evoked potential (HEP) at F8 and C4 channels between PNES and ES, demonstrating HEP amplitude difference between PNES and ES patients across frontal and central areas [17]. Furthermore, a dynamic change in HEP amplitude between interictal and preictal states was observed in the PNES group by HEP amplitude difference between interictal and preictal states in PNES, while the ES group showed no differences in HEP amplitude between states [17]. These findings underscore the need to examine entropy in a spatially resolved, state-dependent framework. The scarcity of evidence on how EEG entropy features evolve across brain states—particularly the transition from interictal (baseline) to preictal (pre-seizure)—further limits current understanding. Most prior work treats EEG as a static signal, overlooking temporal fluctuations in brain complexity that may be critical for accurate diagnosis.

These limitations motivated the current study, which aims to conduct a comprehensive, subject-level analysis of EEG entropy features for PNES versus ES classification using a wide range of machine learning models. Specifically, we applied different entropy measures—spanning conditional, ordinal, symbolic, frequency, fuzzy, phase, and time–frequency domains, thereby providing a broad assessment of neural irregularity—from EEG signals from patients diagnosed with PNES or ES. Unlike previous studies that combined different entropy metrics into a single feature set, we evaluated each entropy measure individually to determine its independent diagnostic value. To address spatial and temporal dynamics, we assessed how EEG entropy features vary across scalp regions and between preictal and interictal states. In addition, we employed permutation importance ranking to filter out redundant EEG channels and identify the most critical electrodes contributing to PNES–ES classification, thereby enhancing interpretability and reducing channel redundancy. Our central hypothesis is that entropy measures capture meaningful differences in brain signal irregularity between PNES and ES, and that these differences manifest across both spatial and temporal dimensions. To test this, we performed both group-level statistical analyses and machine-learning-based classification within a subject-independent validation framework, ensuring robust and clinically meaningful generalisation.

The remainder of this paper is structured as follows. Section 2 describes the materials and methods, including data collection, EEG pre-processing, entropy feature extraction, parameter optimisation, subject-level classification, and statistical analyses. Section 3 presents the results, covering hyperparameter selection, demographic comparisons, statistical analyses of entropy features, permutation importance ranking, and classification performance. Section 4 provides a detailed discussion of the findings in the context of existing literature, and Section 5 concludes the paper by summarising the main contributions and outlining directions for future research.

## 2. Materials and Methods

### 2.1. Data Collection

The study used a database collected at St George’s Hospital in London, comprising EEG recordings from 74 patients diagnosed with either PNES or ES. Signals were acquired using a Natus NeuroWorks system (Natus Medical Incorporated, Middleton, WI, USA) with an EEG32 headbox and silver/silver chloride cup electrodes placed according to the international 10–20 system. Recordings were referenced to Cz–Pz, sampled at either 256 Hz or 1024 Hz, and band-pass filtered with a 0.5–70 Hz Butterworth filter to reduce low-frequency drifts and high-frequency noise. From the 32 available EEG channels, we selected seventeen common channels (F7, T3, T5, O1, F3, C3, P3, Fz, Cz, F8, T4, T6, O2, F4, C4, P4, Pz) that provided broad coverage of frontal, central, temporal, parietal, and occipital regions, while excluding auxiliary sensors such as EOG, EMG, ECG, and reference channels that are not directly relevant to cortical activity.

The cohort included 46 PNES patients (14 males, 32 females; age range: 17–59 years, mean 36.78 ± 11.51) and 28 ES patients (18 males, 10 females; age range: 19–79 years, mean 39.60 ± 14.06). Each patient was diagnosed by at least two epilepsy specialists through video-EEG monitoring, and exclusion criteria comprised comorbid epileptic and functional non-epileptic seizures. Interictal and preictal EEG epochs were identified by these specialists and, since the length of recordings varied, a fixed one-minute EEG segment was selected per subject per condition (preictal and interictal) to ensure comparability across participants.

### 2.2. EEG Pre-Processing

To standardise analyses and reduce computational load, EEG data were downsampled to 128 Hz using MNE-Python v1.2.1, which performs frequency-domain interpolation with edge padding to minimise spectral leakage [18]. This rate preserves the 0.5–40 Hz frequency content relevant for this study with an adequate Nyquist margin. All downsampled signals were then band-pass filtered between 0.5 and 40 Hz using a zero-phase FIR filter with a Hamming window (order = 845, passband ripple < 0.02 dB, stopband attenuation ≈ 53 dB).

For artefact handling, filtered EEGs were segmented into 1 s, non-overlapping epochs. Short epochs allow the detection of transient artefacts such as eye blinks and muscle bursts with higher temporal precision [19]. EEG epochs with near-flat activity were removed (peak-to-peak amplitude < 1 µV). Remaining epochs were cleaned with the Autoreject algorithm, which learns sensor-specific peak-to-peak rejection thresholds by cross-validation and then repairs trials by spherical-spline interpolation of up to *ρ* “worst” sensors; trials with more than *κ* bad sensors are rejected [20]. The parameters, sensor thresholds, and the pair (*ρ*,*κ*) are estimated from the data via cross-validation without manual tuning. We used the standard Autoreject configuration with EEG channel picks and a fixed random state for reproducibility [20]. After artefact removal, the cleaned data were recombined into longer segments for entropy analysis.

### 2.3. Entropy Feature Extraction

Entropy features were computed from 5 s, non-overlapping, clean EEG epochs. At 128 Hz sampling, each epoch contained 640 samples, providing sufficient data for reliable estimation of entropy features [21]. We selected nine complementary entropy algorithms covering time, frequency, symbolic, phase, and information-theoretic perspectives. SampEn estimates the probability that two patterns of length *m* that are similar within a tolerance *r* remain similar when extended by one sample; here, *m* is the embedding dimension, and *r* is the similarity threshold [7]. FuzzyEn replaces the hard similarity rule with a fuzzy membership function to improve stability in noisy signals, using the same *m* and *r* together with a fuzzy power *n* that controls membership sharpness [22]. PermEn quantifies structural complexity from the diversity of ordinal patterns formed by local rank orderings, parameterised by embedding dimension *m* and an embedding delay *τ* [23]. Dispersion entropy (DispEn) combines amplitude and order information by mapping samples to *c* classes and analysing dispersion patterns formed with embedding dimension *m* [24]. Conditional entropy (CondEn) captures the average uncertainty in the next symbol given the current state in a symbolised sequence, defined by class count *c* and embedding dimension *m* [25]. Phase entropy (PhasEn) summarises the uniformity of instantaneous phase distributions using phase histograms with *k* bins—lower values indicate phase locking and higher values indicate desynchronisation [26]. SpecEn is Shannon entropy of the normalised power spectrum and reflects the dispersion of spectral energy across frequencies [27]. RenEn generalises Shannon entropy via an order parameter α that modulates sensitivity to common versus rare events [28], and Wavelet entropy (WaveEn) evaluates the dispersion of energy across time–frequency scales [29].

For each subject and each entropy type, one entropy value was extracted from every 5 s epoch within a 1 min EEG recording across 17 channels, during both preictal and interictal states. This yielded a 12 × 17 matrix of entropy values for each state. Preictal and interictal epochs were selected independently from separate, clinically defined time periods; they were not assumed to be temporally adjacent. A third, “dynamic” representation was also derived as the element-wise difference between preictal and interictal entropy values. All statistical tests and classification analyses were performed separately for each entropy type and for each of the three conditions. With 74 subjects, stacking the 12 epochs per subject produced a design matrix of size 888 × 17 per entropy and per condition for model development.

### 2.4. Parameter Optimisation for Entropy Measures

Since configuration settings strongly affect the sensitivity and reliability of entropy estimates in short EEG epochs, parameter values were optimised using a leakage-free nested procedure within the training folds of the leave-one-subject-out cross-validation (LOSO-CV). A grid search was conducted to identify the configuration that maximised mean AUC across the inner validation folds. The grids were as follows: SampEn with (*m*, *r*) in {(1, 0.1), (1, 0.2), (2, 0.1), (2, 0.2)} where *r* is expressed as a fraction of the standard deviation of a single epoch; FuzzyEn with the same combinations (*m*, *r*) and fuzzy power *n* = 2; PermEn with *m* ∈ {3, 4, 5}; DispEn with (*m*, *c*) ∈ {(2, 5), (2, 6), (2, 7)}; CondEn with (*m*, *c*) ∈ {(2, 5), (2, 6), (2, 7)}; and PhasEn with *k* ∈ {4, 8, 12} phase bins. A one-sample embedding delay was used to preserve temporal ordering and reduce aliasing risk [30]. Non-tunable measures followed standard implementations: SpecEn from the normalised FFT power spectrum, RenEn with α = 2, and WaveEn based on Morlet wavelet decomposition, which is commonly used in EEG analysis [31]. When analysing the dynamic condition, we kept the input parameters of the different entropies fixed so that differences reflect state contrasts rather than configuration changes.

### 2.5. Subject-Level Classification and Evaluation

Model evaluation used LOSO-CV. In each fold, one subject served as the test set and the remaining 73 subjects formed the training set, yielding training and test shapes of 73 × 12 × 17 and 1 × 12 × 17 per entropy and condition. Features were standardised with z-scores computed on the training data only and applied to the held-out subject to prevent information leakage. To mitigate class imbalance between PNES (46 subjects, label = 1) and ES (28 subjects, label = 0), random undersampling at the subject level equalised the number of subjects per class in the training data; epoch-level resampling was avoided to preserve within-subject structure.

Within each training fold, permutation importance was applied to identify informative electrodes for each entropy measure. A model was trained and validated, and individual channels were permuted independently over ten repetitions. The mean reduction in AUC across the inner five validation folds was taken as the importance score, with larger reductions indicating greater channel importance. Channels showing consistently positive mean importance were retained, thereby reducing the number of electrodes from the original 17 to a smaller subset determined by their importance ranking. The same selected channels were then applied to the held-out test subject to ensure consistent dimensionality reduction. Following class balancing and channel selection, the training feature matrix was reduced from (73 × 12, 17) to (*N* × 12, *C*), where *N* (*N* < 73) is the number of subjects after undersampling and *C* (*C* < 17) is the number of selected channels.

The processed features were used to train kNN, NB, linear discriminant analysis (LDA), LR, SVM, random forest (RF), multilayer perceptron (MLP), and extreme gradient boosting (XGBoost). Hyperparameters for each model were tuned within nested five-fold cross-validation using the mean AUC as the objective, and decision thresholds were calibrated by maximising the geometric mean of sensitivity and specificity. RF was optimised for the number of trees, tree depth, and splitting and leaf criteria; NB for the variance smoothing parameter; MLP for hidden layer size, regularisation strength, and learning rate; XGBoost for tree number and depth, learning rate, and both L1 and L2 regularisation; LR for the inverse regularisation strength; SVM for the penalty parameter (and kernel coefficient in the RBF case); and kNN for the number of neighbours, distance metric, and weighting scheme. Each model was retrained with its optimal hyperparameters and evaluated on the test folds, generating epoch-level predictions for the 12 segments of the held-out subject. These predictions were then combined using a hard-voting rule to yield a single subject-level label. To obtain the final evaluation metrics, the confusion matrices from each test fold were aggregated over all LOSO-CV folds, and the performance metrics were then computed from the combined counts of true positives (*TP*), true negatives (*TN*), false positives (*FP*), and false negatives (*FN*). The estimated performance metrics are balanced accuracy, recall for PNES class (*sensitivity*), recall for the ES class (*specificity*), and F1 score as defined in Equations (1)–(4).

(1) $$Sensitivity=\frac{TP}{\left(TP+FN\right)}$$

(2) $$Specificity=\frac{TN}{\left(TN+FP\right)}$$

(3) $$Balanced\;Accuracy=\frac{\left(Sensitivity+Specificity\right)}{2}$$

(4) $$F1\;Score=\frac{2TP}{2TP+FP+FN}$$

### 2.6. Statistical Analysis

We assessed demographic differences between PNES and ES cohorts to identify potential confounding variables. Age, a continuous variable, was compared using Welch’s *t*-test, which does not assume equal variances between groups. Sex, a categorical variable, was compared using the Chi-square test of independence to examine differences in distribution.

To assess group-level differences in entropy features between patients with epileptic ES and PNES, we used the non-parametric Mann–Whitney U test (*p* < 0.05). This test was chosen due to the non-normal distribution of the entropy features, as verified using the Kolmogorov–Smirnov test for normality. For more interpretable comparisons, U statistics were converted to z-scores. To account for multiple comparisons across EEG channels, we applied the False Discovery Rate (FDR) correction using the Benjamini–Hochberg procedure [32]. In addition, we quantified the effect size (magnitude of group differences) using the rank-biserial correlation coefficient obtained from the U statistic [33]. All statistical analyses were conducted within each state using entropy features estimated with the optimised parameter values.

## 3. Results

### 3.1. Selection of Entropy Hyperparameter

Entropy features with different parameter configurations were evaluated using the average AUC scores of all classifiers across the LOSO-CV folds. The detailed results for all tested settings for each state are reported in Tables S1–S3 of the Supplementary Materials. The selected hyperparameters are: SampEn (*m* = 1, *r* = 0.2), FuzzyEn (*m* = 2, *r* = 0.2), PermEn (*m* = 3), DispEn (*c* = 7), CondEn (*c* = 6), and PhasEn (*k* = 16) for the preictal state; SampEn (*m* = 2, *r* = 0.2), FuzzyEn (*m* = 2, *r* = 0.2), PermEn (*m* = 5), DispEn (*c* = 5), CondEn (*c* = 6), and PhasEn (*k* = 8) for the interictal state; and SampEn (*m* = 2, *r* = 0.1), FuzzyEn (*m* = 2, *r* = 0.1), PermEn (*m* = 5), DispEn (*c* = 6), CondEn (*c* = 6), and PhasEn (*k* = 16) for the dynamic state. These best-performing parameters were then used for all subsequent analyses.

### 3.2. Demographic Comparison Between PNES and ES

The analysis of demographic variables revealed a significant difference in the sex distribution between PNES and ES groups (*χ*^2^(1) = 6.81, *p* < 0.01), with a higher proportion of females in the PNES group (32F/14M) compared to the ES group (10F/18M). However, no significant difference was observed in age between the two cohorts.

### 3.3. Group-Level Statistical Comparisons of Entropy Features

**Figure 1:**
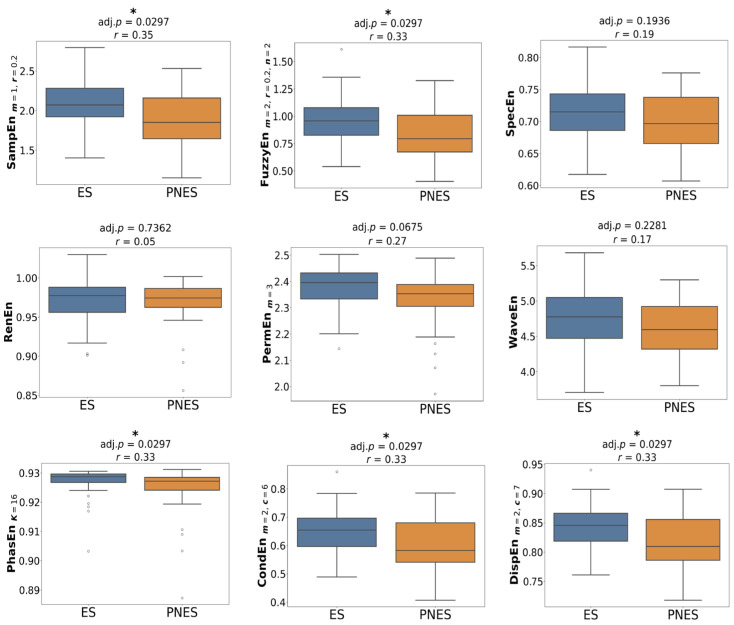
Group-level statistical comparison of entropy features with the best parameters between ES and PNES groups across preictal state. Statistically significant group differences are indicated by asterisks (* *p* < 0.05), with associated *p*-values and effect sizes (*r*) reported above each plot.

**Figure 2:**
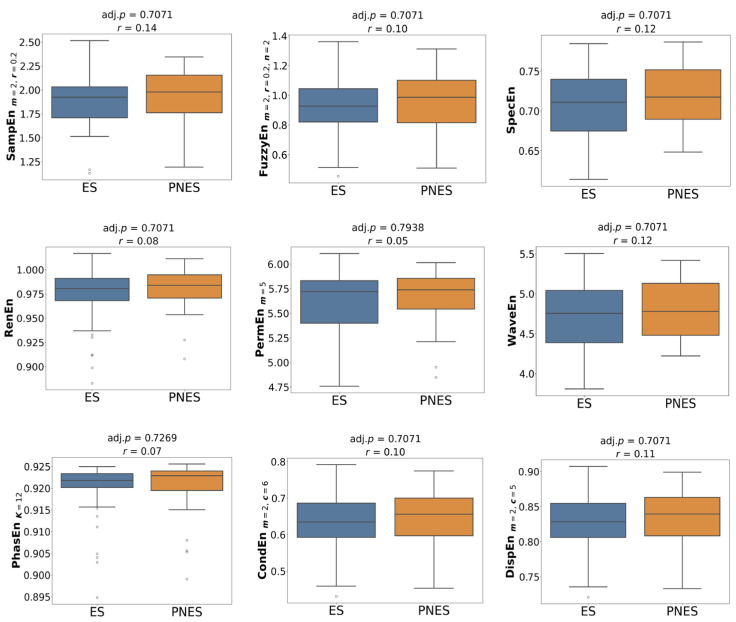
Group-level statistical comparison of entropy features with the best parameters between ES and PNES groups across interictal state, with associated *p*-values *r* reported above each plot.

**Figure 3:**
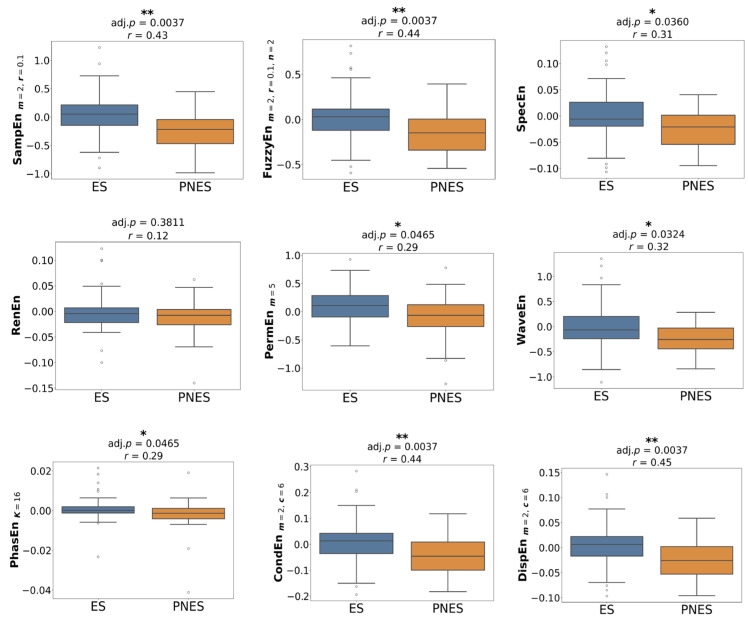
Group-level statistical comparison of entropy features with the best parameters between ES and PNES groups across dynamic state. Statistically significant group differences are indicated by asterisks (* *p* < 0.05, ** *p* < 0.01), with associated *p*-values and *r* reported above each plot.

Figure 1 presents the group comparison of entropy values using optimised parameters. In the preictal state, SampEn, FuzzyEn, CondEn, PhasEn, and DispEn were significantly reduced in the PNES group compared with ES (*p* = 0.03, *r* = 0.33–0.35). Other measures did not reach statistical significance, although lower median entropy values were still observed for PNES. In the interictal state (Figure 2), none of the entropy features differed significantly between groups; however, median values tended to be slightly higher in PNES than in ES. The most pronounced group differences appeared in the dynamic state (Figure 3). All entropy features, except for RenEn, showed significant group differences. SampEn, FuzzyEn, CondEn, and DispEn reached *p* < 0.01 with effect sizes of *r* = 0.43–0.45, while the remaining features reached *p* < 0.05 with effect sizes of *r* = 0.29–0.32. Importantly, dynamic entropy changes in ES were centred around zero, reflecting no consistent difference between interictal and preictal periods, whereas PNES patients exhibited negative entropy shifts, indicating reduced entropy from interictal to preictal states.

### 3.4. Spatial Distribution of Entropy Differences Across the Scalp

**Figure 4:**
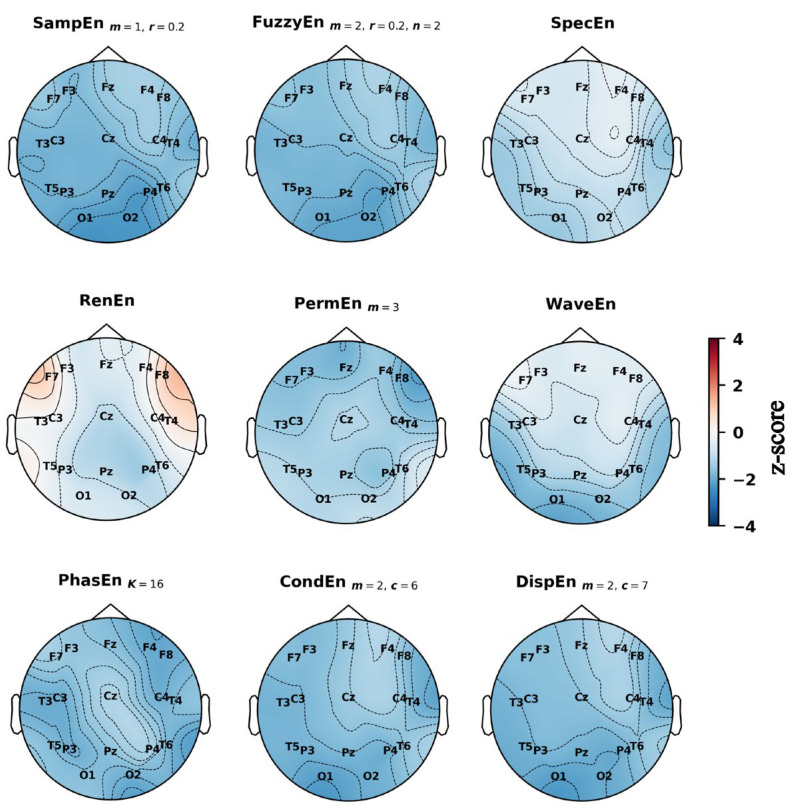
Topographic distribution of group-level *z*-scored entropy values across the scalp during preictal state using the best parameters. Blue indicates lower entropy values in the PNES group relative to ES, while red indicates higher values.

**Figure 5:**
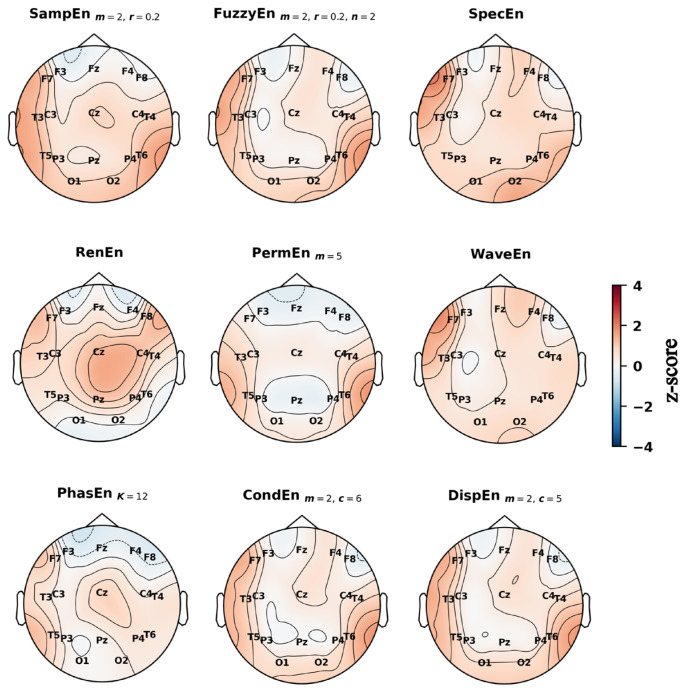
Topographic distribution of group-level *z*-scored entropy values across the scalp during interictal state using the best parameters. Blue indicates lower entropy values in the PNES group relative to ES, while red indicates higher values.

**Figure 6:**
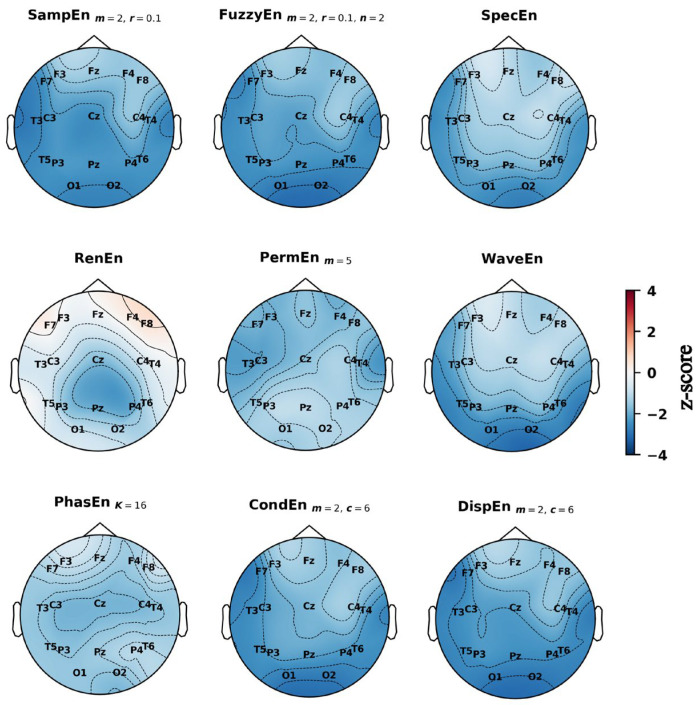
Topographic distribution of group-level *z*-scored entropy values across the scalp during dynamic state using the best parameters. Blue indicates lower entropy values in the PNES group relative to ES, while red indicates higher values.

Topographic maps of z-scored entropy values provided further insight into the spatial distribution of group differences (Figure 4, Figure 5 and Figure 6). In the preictal state (Figure 4), entropy was generally lower in PNES across most electrodes, particularly in the posterior-occipital region (O1, O2). An exception was RenEn, which showed higher values in the frontal sites (F7, F8). In the interictal state (Figure 5), entropy in PNES was higher across several regions: temporal sites (notably T6) for SampEn, FuzzyEn, PermEn, CondEn, and DispEn; the central–parietal midline (Cz, Pz) for RenEn; and the left frontal (F7) for SpecEn and WaveEn. In the dynamic state (Figure 6), spatial differences resembled those observed preictally but were more pronounced. Significant entropy reductions were evident in the posterior-occipital (O1, O2), temporal (T3, T4, T5, T6), and left frontal (F7) regions across multiple measures (SampEn, FuzzyEn, SpecEn, WaveEn, CondEn, DispEn). By contrast, PhasEn and PermEn showed only mild reductions across the scalp, while RenEn exhibited a heterogeneous pattern, with decreases in the central–parietal midline (Cz, Pz) and increases in the frontal cortex.

### 3.5. Permutation Importance Ranking

**Figure 7:**
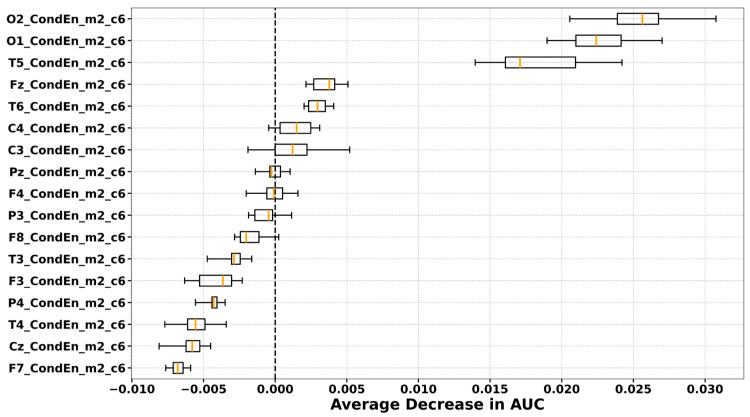
Permutation channel importance for distinguishing PNES from ES during preictal state, assessed via the average decrease in AUC across subjects. Each boxplot displays the distribution of importance scores for individual EEG channels, computed by permuting the values of each channel and observing the resulting change in model AUC. Results are shown for the CondEn with best parameters and XGBoost resulted in the highest mean cross-validation AUC (0.6214).

**Figure 8:**
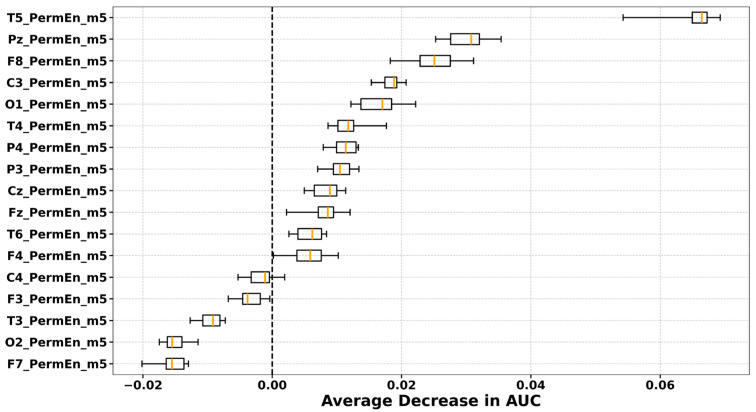
Permutation channel importance for distinguishing PNES from ES during interictal state, assessed via the average decrease in AUC across subjects. Each boxplot displays the distribution of importance scores for individual EEG channels, computed by permuting the values of each channel and observing the resulting change in model AUC. Results are shown for the PermEn with best parameters and LDA resulted in the highest mean cross-validation AUC (0.6012).

**Figure 9:**
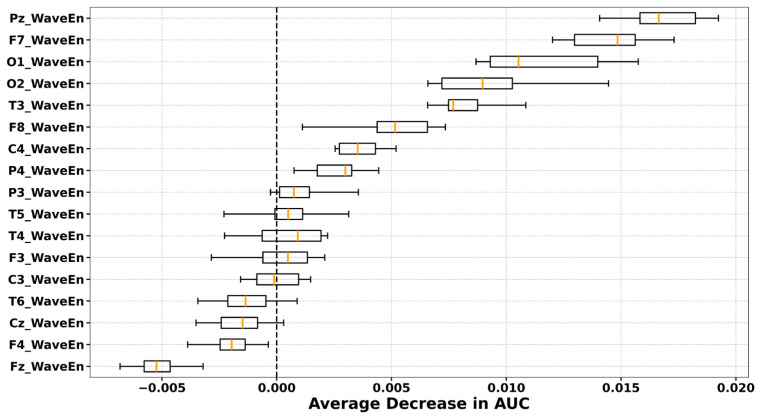
Permutation channel importance for distinguishing PNES from ES during dynamic state, assessed via the average decrease in AUC across subjects. Each boxplot displays the distribution of importance scores for individual EEG channels, computed by permuting the values of each channel and observing the resulting change in model AUC. Results are shown for the WaveEn and SVM resulted in the highest mean cross-validation AUC (0.6279).

Permutation importance values are model-dependent and vary across classifiers, entropy features, and brain states. To ensure comparability, we plotted the importance rankings derived from the classifier–entropy pair that achieved the highest mean AUC under optimised hyperparameters across LOSO-CV folds. The full AUC results for all classifiers and entropy features in the preictal, interictal, and dynamic states are provided in the Supplementary Materials (Tables S4–S6). The highest mean AUCs were obtained with XGBoost using CondEn in the preictal state (AUC = 0.6214), LDA using PermEn in the interictal state (AUC = 0.6012), and SVM using WaveEn in the dynamic state (AUC = 0.6279). The average permutation importance values across all channels for these best-performing classifier–entropy combinations are depicted in Figure 7 (preictal), Figure 8 (interictal), and Figure 9 (dynamic).

In the preictal state, channels O1, O2, T5, T6, and Fz were selected in every fold, with O1, O2, and T5 emerging as the most influential. In the interictal state, a broader range of channels contributed, but T5 consistently ranked as the most important across all LOSO-CV folds. In the dynamic state, consistently selected electrodes were located over frontal (F7, F8), central (C4), temporal (T3), parietal (Pz, P4), and occipital (O1, O2) regions, although their relative importance varied across folds.

### 3.6. Classification Performance

The binary classification performance of all entropy features was evaluated on unseen subjects using balanced accuracy, F1 score, sensitivity, and specificity. Detailed results are reported in the Supplementary Materials (Table S4: preictal, Table S5: interictal, Table S6: dynamic).

**Figure 10:**
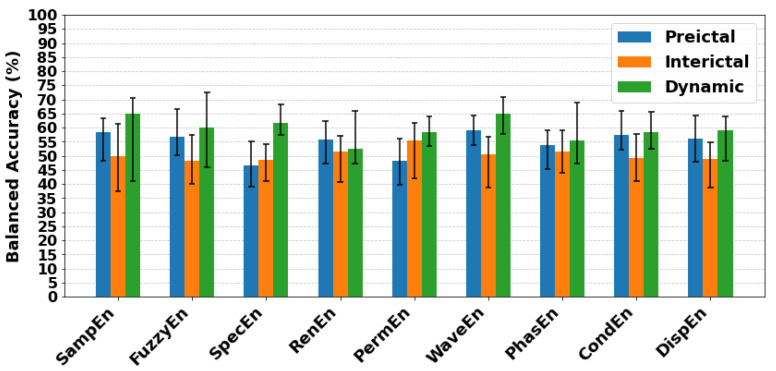
Mean balanced accuracy for each entropy feature (SampEn, FuzzyEn, SpecEn, RenEn, PermEn, WaveEn, PhasEn, CondEn, DispEn) across preictal, interictal, and dynamic states. Results were averaged over all classifiers (kNN, NB, LDA, LR, SVM, RF, XGBoost, MLP). Error bars indicate the standard deviation of balanced accuracy across classifiers.

For an overall comparison, Figure 10 summarises the mean and standard deviations of balanced accuracy across all classifiers for each entropy feature and brain state. Mean balanced accuracy values for all features were higher in the dynamic state than in the preictal or interictal states. WaveEn (64.99%) and SampEn (64.82%) resulted the best mean balanced accuracy, while the highest overall performance was achieved by FuzzyEn with SVM (balanced accuracy 72.42%, F1 score 77.78%, sensitivity 74.47%, specificity 70.37%).

When comparing preictal and interictal conditions, preictal classification generally outperformed interictal. For preictal data, WaveEn and SampEn achieved mean balanced accuracies of 59.19% and 58.39%. The strongest individual preictal result was obtained with FuzzyEn using XGBoost with a balanced accuracy of 66.63%, F1 score of 79.21% and sensitivity of 85.11%, but specificity was below chance level (48.15%). In the interictal state, the best result was achieved by PermEn with SVM (balanced accuracy = 61.51%), which showed acceptable specificity (74.07%) but poor sensitivity (48.94%) and a modest F1 score (59.74%).

**Figure 11:**
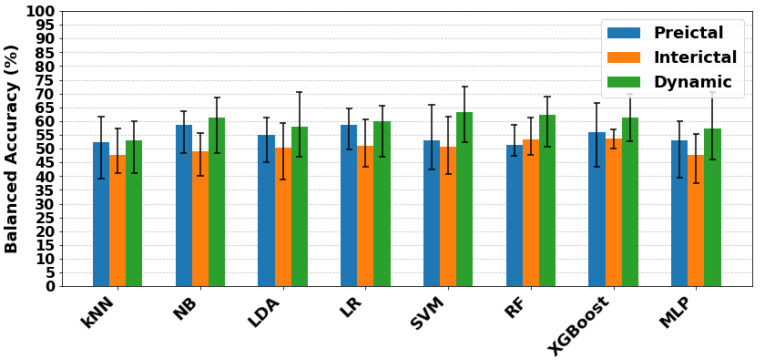
Mean balanced accuracy for each classifier (kNN, NB, LDA, LR, SVM, RF, XGBoost, MLP) across preictal, interictal, and dynamic states. Results were averaged over all features (SampEn, FuzzyEn, SpecEn, RenEn, PermEn, WaveEn, PhasEn, CondEn, DispEn). Error bars indicate the standard deviation of balanced accuracy across entropy features.

Classifier-level comparisons are shown in Figure 11, where the mean balanced accuracy across all entropy features is displayed by state. Consistent with entropy-level results, classifiers performed best in the dynamic state, with SVM achieving the highest mean balanced accuracy (63.15%). Preictal performance was generally higher than interictal for most classifiers, except RF. In this state, LR and NB performed similarly, both reaching mean balanced accuracy of 58.52% and 58.57%, respectively.

## 4. Discussion

This study investigated the utility of entropy features extracted from EEG for distinguishing between PNES and ES across different brain states. A comprehensive set of analyses—including group-level statistics, topographic mapping, channel-wise importance estimation, and classification performance—revealed converging evidence that entropy dynamics between states offer overall superior discriminative power compared to static features alone.

Group-level statistical comparisons demonstrated that the strongest differences between PNES and ES emerged in the dynamic state, defined as the change in entropy from interictal to preictal periods. In this condition, a significant reduction in entropy changes in PNES group appeared for almost all entropy measures. Particularly, the difference became more significant for FuzzyEn, SampEn, CondEn, and DispEn. This suggests that PNES is characterised by a reduction in EEG unpredictability as the seizure approaches, whereas ES shows no consistent change from interictal to preictal—some subjects showed increased entropy, others decreased, and many remained stable—reflecting more heterogeneous or stable neural dynamics. These results align with a recent study by Elkommos et al., which reported a significant decrease in HEP amplitudes from interictal to preictal periods in PNES patients, but not in those with ES [17]. Together, the evidence suggests a broader decline in neural responsiveness or signal complexity preceding functional episodes, adding insight into the preictal mechanisms that may underlie the generation of functional seizures. During the interictal period, no significant group differences were observed across any entropy method. In the preictal period, SampEn, FuzzyEn, CondEn, PhasEn, and DispEn showed significant group differences, with lower entropy values observed in PNES compared to ES. However, their effect sizes were smaller than those observed in the dynamic condition, underscoring the added diagnostic value of capturing temporal changes in entropy rather than relying on single-state measurements.

Classification results confirmed these findings by showing that dynamic entropy features returned the best generalisation performance across unseen subjects. At the classifier level, SVM showed the most robust performance in the dynamic state, reflecting its ability to exploit non-linear boundaries in temporally varying features. In contrast, during the preictal state, simpler models such as NB and LR achieved better classification across the entropy measures, suggesting that linear or probabilistic approaches may be more effective in discriminating PNES from ES in the preictal period. Across all conditions, preictal performance generally exceeded interictal, but both were outperformed by dynamic state.

Among the entropy families, FuzzyEn stood out as one of the most informative descriptors across states. WaveEn and SampEn also showed statistical differences between PNES and ES not only in the dynamic state but also in the preictal state, indicating that both time–frequency and pattern-regularity perspectives capture state-dependent changes relevant to PNES–ES differentiation. To the best of our knowledge, our work further introduces alternative entropy measures that have not been previously explored in the context of PNES. Notably, WaveEn resulted in the best average balanced accuracy in both preictal and dynamic states, while CondEn and DispEn showed strong statistical differences (*p* < 0.01) in dynamic state. By contrast, PermEn had a lower overall performance but returned the best-performing feature in interictal condition, suggesting it may capture complementary aspects of neural variability during resting conditions.

Our findings are partially in agreement with previous research. Kienitz et al. focused on distinguishing patients with TLE from those with NEEs during the interictal state and reported reduced SampEn in the ES group compared with NEEs [11]. In our analysis, a similar trend of lower SampEn in ES compared with PNES was observed during the interictal state, but this difference did not reach statistical significance. A direct comparison cannot be made as NEEs may not necessarily arise from psychogenic origins and their analysis was restricted to a specific epilepsy subtype. The discriminative value of FuzzyEn in our data set is also supported by Tibdewal et al., who identified FuzzyEn as a strong feature for differentiating ES from non-epileptic EEG [15]. Conversely, while RenEn has previously been reported as a promising classifier [6,8], it did not emerge as useful in our analysis, being the only entropy feature that showed no significant group differences in the dynamic state and contributed little to classification. For SpecEn, our results partly resonate with earlier PNES studies [6,8,10]. While it did not differentiate groups in preictal and interictal states, we observed significant effects in the dynamic state, alongside improved classification performance. Results for PermEn extend previous findings by Gasparini et al. and Lo Giudice et al., who reported no significant differences in PNES vs. HC and PNES vs. ES, respectively [13,14]. In our study, PermEn was significant in the dynamic state, and importantly, it provided the best classification performance in the interictal condition.

Our topographic and permutation analyses provided complementary insights into the spatial characteristics of PNES versus ES differences. In the interictal state, group differences appeared more focal, with entropy values in PNES rising relative to ES at selected regions. Topographic maps highlighted increases in entropy values at the right temporal site (T6) and the left frontal site (F7), while permutation importance consistently identified T5 as the most discriminative channel. In the preictal state this focal pattern shifted to a widespread reduction in entropy in PNES, affecting nearly all electrodes. The decrease was most pronounced at the posterior–occipital electrodes (O1, O2), which were also ranked among the most important channels in the permutation analysis, alongside T5, T6, and Fz. These interictal rises followed by preictal reductions created stronger group differences overall, particularly in the posterior regions (O1, O2, Pz), as well as in the temporal areas and the left frontal site (F7). These same regions overlapped with the most important electrodes identified during the dynamic state, where permutation analysis highlighted Pz, O1, O2, F7, and T3 as the top contributors.

Only a limited number of studies have investigated the spatial distribution of entropy in PNES and ES, and our work adds to this scarce evidence by characterising state-dependent entropy changes from interictal to preictal periods. A comparable approach was employed by Vega-Zelaya et al., who computed SpecEn as percentage ictal changes normalised to a basal state and reported reduced values over the frontal, parieto-occipital, and temporal regions in patients experiencing BPA [10]. Their findings resonate with our observation that entropy differences between PNES and ES are most prominent across posterior–occipital, temporal, and frontal regions, particularly when analysed within the state difference. This parallel suggests that reductions in entropy within these cortical areas may represent a more general marker of altered neural complexity preceding non-epileptic or atypical paroxysmal events.

Further insight into spatial dynamics was provided by Lo Giudice et al., who applied a CNN model to classify PNES vs. ES patients using interictal EEG and then analysed latent feature representations using PermEn [14]. They found no significant group differences in PermEn at the raw EEG input level, consistent with our statistical findings. However, entropy differences emerged in central and parietal regions at the first convolutional layer (Conv1) and became more widespread at Conv2 [14]. These areas partially overlapped with those found in our analysis, where RenEn showed higher—but not statistically significant—entropy in PNES over the central and parietal regions during the interictal condition. These converging spatial findings suggest that even when statistical separability is limited, signal complexity in specific brain areas may hold diagnostic value.

Adding further to the evidence base, Allendorfer et al. used resting-state fMRI to compute SampEn across large-scale functional networks in patients with TBI, TBI + ES, and TBI + PNES [16]. Their study found that SampEn in the frontoparietal network is lower compared to other networks. Although their data and cohort differ from ours, their findings support the notion that PNES is associated with unique spatial alterations in entropy, particularly resting states.

Taken together, the literature reveals inconsistencies regarding the spatial patterns for distinguishing PNES from ES, which may arise from differences in patient cohorts, data modalities (EEG vs. fMRI), entropy metrics, or the brain states examined (interictal, preictal, ictal). Our results show that these spatial patterns are state-dependent: entropy differences in PNES became more focal during the interictal period, localising mainly to the temporal lobes (T5, T6) and left frontal cortex (F7), but shifted to a widespread reduction across the scalp during the preictal period, most pronounced over the posterior–occipital sites (O1, O2). When dynamic changes from interictal to preictal were evaluated, the strongest group differences were observed at electrodes over the posterior–occipital, temporal, central-parietal (Pz), and left frontal (F7) regions. These findings suggest that PNES cannot be localised to a single cortical region; rather, their discriminative features emerge from a distributed pattern of altered entropy that reorganises across states. A meta-analysis identified grey matter abnormalities in the left temporal lobe (Brodmann area 21) in PNES compared with HC [34], and there are reports of temporal lobe abnormalities in individual PNES studies [35,36]. At the same time, functional studies such as Elkommos et al. observed right-lateralised reductions in dynamic HEP amplitudes over frontocentral electrodes (F8, C4) [17], indicating that both hemispheres and multiple cortical hubs can contribute. Together, these findings point to PNES as a disorder underpinned by multifocal network disruption, with the relative importance of temporal, posterior, frontal, and central regions depending on the brain state examined.

This study has several limitations that should be considered when interpreting the findings. First, although entropy features demonstrated promising discriminative power, the overall classification performance remained moderate, suggesting that entropy alone may not fully capture the complexity of differentiating PNES from ES using EEG signals. Future work could explore combining entropy with complementary measures such as frequency-domain features or non-linear complexity indices (e.g., fractal dimensions) to build more comprehensive models of brain dynamics. Second, while this study focused on entropy features with conventional machine learning classifiers, recent studies have applied DL models (e.g., CNNs, autoencoders) directly to EEG for PNES versus ES classification [13,14,37,38]. Future work could extend those models using Long short-term memory (LSTM) and Transformers to capture richer temporal and spatial dynamics. Third, our analysis revealed a significant gender imbalance, with a higher proportion of female patients in the PNES group. This demographic difference may have introduced bias into the results, and incorporating demographic variables explicitly into the models may help to mitigate such effects and improve model performance. Fourth, we did not explore multiscale entropy approaches, which can capture neural irregularity across different temporal and spectral resolutions and may provide additional discriminatory power. Fifth, although our spatial analysis identified meaningful topographic patterns, the use of standard EEG with limited electrode coverage constrains spatial resolution; replication with high-density EEG would allow for more precise localisation and validation of critical regions. Finally, our sample size, while comparable to other PNES–ES studies, remains modest, and external validation using larger, multi-centre datasets is needed to confirm the robustness of our findings.

## 5. Conclusions

This study systematically evaluated multiple entropy measures applied to EEG signals to distinguish PNES from ES across interictal, preictal, and dynamic states using subject-independent validation. The results showed that dynamic entropy features, reflecting changes from interictal to preictal periods, provided superior discriminative power compared to static measures. Among the evaluated features, FuzzyEn, SampEn, and WaveEn for preictal and dynamic states and PermEn for interictal state outperformed other features. Spatial analyses revealed state-dependent patterns. In the interictal state, group differences were focal, mainly in the temporal and frontal regions. In the preictal state, the differences became more uniform across electrodes, with PNES exhibiting lower entropy than ES. The most pronounced group differences emerged at posterior–occipital, temporal, frontal, and central–parietal regions in the dynamic state: entropy changes from interictal to preictal were significant in PNES, showing a decrease across this transition, whereas no significant within-state change was observed for ES. Although classification performance was moderate, these findings highlight the potential of entropy analysis of the EEG as a complementary tool for PNES–ES differentiation and suggest that future work should combine entropy with additional EEG features, apply multiscale methods, and validate results using high-density EEG and larger multi-centre data sets to enhance generalisability and clinical utility.

## Figures and Tables

**Table 1:** Comparison of entropy-related studies focused on seizure context based on EEG analysis and machine learning. (Please refer to the list of abbreviations for all acronyms used in this table.)

Authors	Cohort Characteristics	EEG Recording	Entropy Measure(s)	Additional Features	Analysis Approach	Classifiers	Results	Notes
[9]	4 ES, 7 PNES	Ictal EEG	ShEn, LogEn	Time- and frequency- domain features	Feature selection (ReliefF)	NB, SVM, RF, kNN	Accuracy: 95% (NB)	Entropy not top-ranked.
[15]	5 ES, 5 non-ES	Ictal and interictal EEG	ShEn, RenEn, ApEn, SampEn, FuzzyEn	-	Statistical comparison	-	FuzzyEn: stronger differences.	Epoch-wise classification.
[8]	20 PNES, 20 ES	Ictal EEG	ShEn, SpecEn, RenEn	HFD, KFD	Feature Selection (ICA)	SVM, RF, DT, GBM.	Accuracy: 95.0%; SpecEn/RenEn most important.	Subject-wise 80/20 train/test splits.
[13]	6 PNES, 10 HC	Preictal EEG	ShEn, PermEn	Statistical features of wavelet sub-bands	Interpretability of DL model; Statistical comparison	SAE, LDA, QDA, SVM	Accuracy: 86.5% (SAE); entropy decreases with depth.	Entropy used for interpretation, no significant group differences.
[10]	15 ES, 21 BPA	Ictal EEG	SpecEn	Power spectrum in EEG bands	Statistical comparison	-	SpecEn dropped in BPA group.	Ictal bursts normalised to pre/post.
[12]	ES, non-ES	Ictal and interictal EEG	SampEn	HFD	Statistical comparison	SVM	Accuracy: 89.8%; SampEn lower in epilepsy.	Epoch-wise classification
[6]	68 PNES, 32 ES	Preictal and interictal EEG	ApEn, SampEn, SpecEn, RenEn, SVDEn, WaveletEn	-	Feature selection (PCA)	SVM, RF, kNN, GBM	Accuracy: 95.0% (kNN); RenEn best feature.	Epoch-wise 10—fold CV
[14]	18 PNE, 18 ES	Interictal EEG	PermEn	CNN: wavelet sub-bands; ML: statistical features of sub-bands.	Interpretability of DL model	CNN, MLP, SVM, QDA, LDA	Accuracy: 94.4% (CNN); deeper layers stronger PermEn differences.	LOSO-CV; entropy only used for model interpretability.
[11]	27 TLE, 24 NEE	Interictal EEG	SampEn	Delta power	Statistical comparison	LR	AUC: SampEn 0.76; Delta 0.5.)	One ES type only

## Data Availability

The data used in this study were provided by St George’s Hospital and are not publicly available due to ethical and privacy restrictions.

## Supplementary Materials

The following supporting information can be downloaded at: https://www.mdpi.com/article/10.3390/e27101044/s1, Table S1: Average AUC scores of classification models using different entropy features under various hyperparameter settings in the preictal state. For each entropy feature, the hyperparameter configuration that achieved highest average AUC is highlighted in bold. Table S2: Average AUC scores of classification models using different entropy features under various hyperparameter settings in the interictal state. For each entropy feature, the hyperparameter configuration that achieved highest average AUC is highlighted in bold. Table S3: Average AUC scores of classification models using different entropy features under various hyperparameter settings in the dynamic state. For each entropy feature, the hyperparameter configuration that achieved highest average AUC is highlighted in bold. Table S4: Average performance of classifiers in the preictal state using entropy features with optimal hyperparameter settings. Table S5: Average performance of classifiers in the interictal state using entropy features with optimal hyperparameter settings. Table S6: Average performance of classifiers in the dynamic state using entropy features with optimal hyperparameter settings.

## Abbreviations

EEG	Electroencephalogram	KFD	Katz fractal dimension
ECG	Electrocardiogram	DL	Deep learning
ES	Epileptic seizures	ML	Machine learning
PNES	Psychogenic non-epileptic seizures	CNN	Convolutional neural network
HC	Healthy control	SAE	Stacked autoencoder
BPA	Bursts of periodic activity	SVM	Support vector machine
TLE	Temporal lobe epilepsy	GBM	Gradient boosting machine
NEE	Non-epileptic episodes	XGBoost	eXtreme Gradient Boosting
ShEn	Shannon entropy	LR	Logistic regression
ApEn	Approximate entropy	DT	Decision tree
SampEn	Sample entropy	RF	Random forest
FuzzyEn	Fuzzy entropy	kNN	k-Nearest Neighbours
SpecEn	Spectral entropy	LDA	Linear discriminant analysis
SVDEn	Singular value decomposition entropy	QDA	Quadratic discriminant analysis
LogEn	Logarithmic energy entropy	MLP	Multi-layer perceptron
WaveEn	Wavelet entropy	NB	Naïve Bayes
PermEn	Permutation entropy	AUC	Area under the curve
RenEn	Rényi entropy	PCA	Principal component analysis
CondEn	Conditional entropy	ICA	Imperialist competitive algorithm
PhasEn	Phase entropy	FFT	Fast Fourier Transform
DispEn	Dispersion entropy	LOSO-CV	Leave-one-subject-out cross-validation
HFD	Higuchi fractal dimension	LSTM	Long short-term memory
