# Supplementary material for: EEG Complexity Analysis of Psychogenic Non-Epileptic and Epileptic Seizures Using Entropy and Machine Learning
Source: Entropy (Basel). 2025 Oct 7;27(10):1044. doi: 10.3390/e27101044 (PMC12564079; doi:10.3390/e27101044)
Supplement: Supplementary file 1 [file entropy-27-01044-s001.zip › entropy-3837146-supplementary.pdf]

Supplementary Materials

Table S1. Average AUC scores of classification models using different entropy features under various hyperparameter settings in the preictal state. For each entropy feature, the hyperparameter configuration that achieved the highest average AUC is highlighted in bold.

Feature	kNN	NB	LDA	LR	SVM	RF	MLP	XGBoost	Mean ± STD
SampEn (m=1, r=0.1)	0.5864	0.6084	0.5301	0.5882	0.5799	0.5951	0.5626	0.5923	0.5804 ± 0.024
**SampEn (m=1, r=0.2)**	0.5990	0.6078	0.5293	0.5889	0.5807	0.5924	0.5566	0.5890	**0.5805 ± 0.026**
SampEn (m=2, r=0.1)	0.5681	0.5990	0.5057	0.5682	0.5443	0.5897	0.4932	0.5898	0.5573 ± 0.040
SampEn (m=2, r=0.2)	0.5693	0.5986	0.4938	0.5618	0.5390	0.5585	0.5563	0.5658	0.5554 ± 0.030
FuzzyEn (m=1, r=0.1)	0.5863	0.6021	0.5154	0.5752	0.4760	0.5990	0.5344	0.5791	0.5584 ± 0.045
FuzzyEn (m=1, r=0.2)	0.5803	0.5987	0.5138	0.5717	0.4675	0.6065	0.5576	0.5804	0.5596 ± 0.047
FuzzyEn (m=2, r=0.1)	0.5805	0.6004	0.5132	0.5729	0.5786	0.5837	0.5413	0.5912	0.5702 ± 0.029
**FuzzyEn (m=2, r=0.2)**	0.5745	0.5912	0.5144	0.5755	0.5824	0.5882	0.5662	0.5803	**0.5716 ± 0.024**
**PermEn (m=3)**	0.5322	0.5833	0.5035	0.5492	0.5446	0.5790	0.4922	0.5498	**0.5417 ± 0.032**
PermEn (m=4)	0.5108	0.5891	0.4991	0.5400	0.5385	0.5570	0.5039	0.5077	0.5308 ± 0.031
PermEn (m=5)	0.5225	0.5883	0.5024	0.5499	0.5480	0.5523	0.5009	0.5343	0.5373 ± 0.029
DispEn (c=5)	0.5924	0.5950	0.5057	0.5662	0.4686	0.5930	0.5218	0.5746	0.5522 ± 0.048
DispEn (c=6)	0.5960	0.5960	0.5062	0.5672	0.4769	0.6069	0.5210	0.5737	0.5555 ± 0.048
**DispEn (c=7)**	0.5800	0.5989	0.5044	0.5698	0.5746	0.6123	0.5546	0.5719	**0.5708 ± 0.032**
CondEn (c=5)	0.5861	0.6058	0.5626	0.6026	0.5986	0.6069	0.5550	0.5998	0.5897 ± 0.020
**CondEn (c=6)**	0.6003	0.6051	0.5647	0.6017	0.6035	0.6151	0.5684	0.6214	**0.5975 ± 0.020**
CondEn (c=7)	0.6085	0.6089	0.5715	0.6062	0.4929	0.6169	0.5304	0.6043	0.5800 ± 0.045
PhasEn (k=4)	0.5244	0.5722	0.5722	0.5898	0.4937	0.5526	0.5095	0.5579	0.5465 ± 0.034
PhasEn (k=8)	0.5387	0.5730	0.5816	0.5914	0.5858	0.5842	0.4999	0.5679	0.5653 ± 0.031
PhasEn (k=12)	0.5361	0.5536	0.5643	0.5936	0.5869	0.5771	0.5072	0.5884	0.5634 ± 0.030
**PhasEn (k=16)**	0.5669	0.5636	0.5725	0.5966	0.5995	0.5661	0.5324	0.5780	**0.5719 ± 0.021**

Table S2. Average AUC scores of classification models using different entropy features under various hyperparameter settings in the interictal state. For each entropy feature, the hyperparameter configuration that achieved the highest average AUC is highlighted in bold.

Feature	kNN	NB	LDA	LR	SVM	RF	MLP	XGBoost	Mean ± STD
SampEn (m=1, r=0.1)	0.5202	0.5489	0.5414	0.5439	0.4733	0.5370	0.4869	0.5768	0.5286 ± 0.034
SampEn (m=1, r=0.2)	0.5122	0.5443	0.5416	0.5408	0.4726	0.5455	0.4816	0.5691	0.5260 ± 0.034
SampEn (m=2, r=0.1)	0.5328	0.5502	0.5562	0.5572	0.4672	0.5455	0.5180	0.5414	0.5336 ± 0.030
**SampEn (m=2, r=0.2)**	0.5192	0.5637	0.5491	0.5510	0.4656	0.5504	0.4898	0.5798	**0.5340 ± 0.039**
FuzzyEn (m=1, r=0.1)	0.5210	0.5420	0.5416	0.5429	0.4828	0.5494	0.4759	0.5659	0.5277 ± 0.034
FuzzyEn (m=1, r=0.2)	0.5280	0.5400	0.5418	0.5430	0.4827	0.5445	0.4695	0.5692	0.5273 ± 0.032
FuzzyEn (m=2, r=0.1)	0.5299	0.5482	0.5393	0.5388	0.4751	0.5489	0.4840	0.5676	0.5290 ± 0.035
**FuzzyEn (m=2, r=0.2)**	0.5376	0.5490	0.5359	0.5361	0.4779	0.5500	0.4781	0.5787	**0.5304 ± 0.035**
PermEn (m=3)	0.5417	0.5083	0.5787	0.5865	0.4835	0.5516	0.5574	0.5586	0.5458 ± 0.047
PermEn (m=4)	0.5495	0.5068	0.6028	0.5958	0.4776	0.5609	0.4904	0.5679	0.5440 ± 0.054
**PermEn (m=5)**	0.5670	0.5055	0.6146	0.6086	0.4791	0.5573	0.4827	0.5822	**0.5496 ± 0.054**
**DispEn (c=5)**	0.5370	0.5464	0.5471	0.5453	0.4796	0.5431	0.4954	0.5766	**0.5338 ± 0.031**
DispEn (c=6)	0.5248	0.5419	0.5467	0.5455	0.4771	0.5438	0.4845	0.5673	0.5290 ± 0.032
DispEn (c=7)	0.5231	0.5429	0.5422	0.5410	0.4816	0.5445	0.4878	0.5627	0.5282 ± 0.029
CondEn (c=5)	0.5040	0.5038	0.5134	0.5145	0.4912	0.5272	0.4734	0.5285	0.5070 ± 0.018
**CondEn (c=6)**	0.5067	0.5069	0.5061	0.5084	0.4944	0.5505	0.4916	0.5493	**0.5142 ± 0.023**
CondEn (c=7)	0.5184	0.5072	0.5137	0.5118	0.4931	0.5328	0.4896	0.5340	0.5126 ± 0.016
PhasEn (k=4)	0.5201	0.5041	0.5108	0.5101	0.4964	0.5357	0.4791	0.5060	0.5078 ± 0.017
**PhasEn (k=8)**	0.5221	0.5121	0.4930	0.4929	0.4979	0.5276	0.4940	0.5302	**0.5087 ± 0.016**
PhasEn (k=12)	0.5085	0.5069	0.4886	0.4895	0.4844	0.5275	0.4846	0.5007	0.4988 ± 0.015
PhasEn (k=16)	0.5095	0.5123	0.4857	0.4856	0.4754	0.5335	0.4876	0.5054	0.4994 ± 0.019

Table S3. Average AUC scores of classification models using different entropy features under various hyperparameter settings in the dynamic state. For each entropy feature, the hyperparameter configuration that achieved the highest average AUC is highlighted in bold.

Feature	kNN	NB	LDA	LR	SVM	RF	MLP	XGBoost	Mean ± STD
SampEn (m=1, r=0.1)	0.5541	0.6112	0.5653	0.6057	0.5398	0.5980	0.5403	0.5915	0.5757 ± 0.029
SampEn (m=1, r=0.2)	0.5550	0.6127	0.5624	0.6050	0.5501	0.6012	0.5709	0.6007	0.5823 ± 0.025
**SampEn (m=2, r=0.1)**	0.5689	0.6143	0.5673	0.6045	0.6099	0.5947	0.5710	0.6072	**0.5922 ± 0.020**
SampEn (m=2, r=0.2)	0.5604	0.6149	0.5589	0.6018	0.5884	0.5824	0.5627	0.6071	0.5846 ± 0.022
FuzzyEn (m=1, r=0.1)	0.5399	0.6071	0.5569	0.5951	0.5389	0.5918	0.5663	0.5838	0.5725 ± 0.026
FuzzyEn (m=1, r=0.2)	0.5370	0.6027	0.5524	0.5904	0.5579	0.5771	0.5578	0.5829	0.5698 ± 0.022
**FuzzyEn (m=2, r=0.1)**	0.5559	0.6084	0.5631	0.6021	0.5580	0.5837	0.5700	0.5916	**0.5791 ± 0.020**
FuzzyEn (m=2, r=0.2)	0.5535	0.6066	0.5663	0.6003	0.5313	0.5933	0.5726	0.5907	0.5768 ± 0.026
PermEn (m=3)	0.5430	0.5934	0.5368	0.5947	0.5940	0.5812	0.5543	0.5717	0.5711 ± 0.024
PermEn (m=4)	0.5450	0.5983	0.5469	0.6023	0.6058	0.5668	0.5721	0.5850	0.5778 ± 0.024
**PermEn (m=5)**	0.5348	0.5994	0.5560	0.6086	0.6079	0.5883	0.5599	0.5894	**0.5805 ± 0.027**
DispEn (c=5)	0.5593	0.6054	0.5561	0.5934	0.5333	0.5856	0.5575	0.5806	0.5714 ± 0.024
**DispEn (c=6)**	0.5509	0.6045	0.5548	0.5948	0.5473	0.5877	0.5685	0.5884	**0.5746 ± 0.022**
DispEn (c=7)	0.5531	0.6053	0.5568	0.5947	0.5184	0.5873	0.5615	0.5902	0.5709 ± 0.029
CondEn (c=5)	0.5490	0.6097	0.5674	0.6018	0.5328	0.5898	0.5593	0.5876	0.5747 ± 0.027
**CondEn (c=6)**	0.5679	0.6133	0.5781	0.6073	0.5346	0.5954	0.5774	0.5913	**0.5832 ± 0.027**
CondEn (c=7)	0.5444	0.6120	0.5687	0.6039	0.5282	0.5934	0.5638	0.5772	0.5739 ± 0.029
PhasEn (k=4)	0.5175	0.5824	0.5710	0.5728	0.5555	0.5619	0.4779	0.5523	0.5489 ± 0.035
PhasEn (k=8)	0.5412	0.5763	0.5696	0.5727	0.5774	0.5542	0.4813	0.5652	0.5547 ± 0.032
PhasEn (k=12)	0.5410	0.5770	0.5521	0.5541	0.5781	0.5820	0.5584	0.5851	0.5660 ± 0.017
**PhasEn (k=16)**	0.5596	0.5739	0.5617	0.5963	0.5882	0.5859	0.4854	0.5967	**0.5685 ± 0.036**

Table S4. Average performance of classifiers in the preictal state using entropy features with optimal hyperparameter settings.

Classifier	Metric	SampEn	FuzzyEn	SpecEn	RenEn	PermEn	WaveEn	PhasEn	CondEn	DispEn
kNN	AUC	0.5908	0.5804	0.5547	0.5494	0.5235	0.5741	0.5669	0.6003	0.5962
	Bal. Accuracy	56.54%	53.07%	39.01%	57.21%	45.55%	61.51%	45.35%	57.57%	53.86%
	F1 score	70.10%	63.74%	48.84%	38.71%	25.81%	59.74%	49.38%	64.37%	62.92%
	Sensitivity	72.34%	61.70%	44.68%	25.53%	17.02%	48.94%	42.55%	59.57%	59.57%
	Specificity	40.74%	44.44%	33.33%	**88.89%**	74.07%	74.07%	48.15%	55.56%	48.15%
NB	AUC	0.6187	0.6093	0.5543	0.5663	0.5910	0.5805	0.5636	0.6051	0.6072
	Bal. Accuracy	62.37%	60.76%	48.31%	53.86%	56.07%	61.82%	58.94%	61.58%	63.44%
	F1 score	74.23%	68.89%	60.87%	62.92%	77.88%	70.33%	74.51%	74.75%	75.51%
	Sensitivity	76.60%	65.96%	59.57%	59.57%	**93.62%**	68.09%	80.85%	78.72%	78.72%
	Specificity	48.15%	55.56%	37.04%	48.15%	18.52%	55.56%	37.04%	44.44%	48.15%
LDA	AUC	0.5570	0.5522	0.5282	0.5851	0.4921	0.5587	0.5725	0.5647	0.5524
	Bal. Accuracy	61.31%	57.84%	55.16%	49.05%	45.11%	53.82%	58.12%	56.78%	57.84%
	F1 score	72.92%	66.67%	58.54%	50.63%	56.18%	53.85%	68.82%	65.17%	66.67%
	Sensitivity	74.47%	63.83%	51.06%	42.55%	53.19%	44.68%	68.09%	61.70%	63.83%
	Specificity	48.15%	51.85%	59.26%	55.56%	37.04%	62.96%	48.15%	51.85%	51.85%
LR	AUC	0.6075	0.6005	0.5289	0.5863	0.5321	0.5731	0.5966	0.6017	0.5975
	Bal. Accuracy	63.16%	64.50%	52.29%	57.01%	49.65%	58.87%	57.57%	59.46%	64.22%
	F1 score	73.68%	76.77%	64.52%	59.26%	64.58%	60.00%	64.37%	72.16%	75.00%
	Sensitivity	74.47%	80.85%	63.83%	51.06%	65.96%	51.06%	59.57%	74.47%	76.60%
	Specificity	51.85%	48.15%	40.74%	62.96%	33.33%	66.67%	55.56%	44.44%	51.85%
SVM	AUC	0.5858	0.4763	0.5418	0.5873	0.5287	0.5909	0.5995	0.6035	0.5016
	Bal. Accuracy	48.31%	50.47%	42.47%	62.33%	49.65%	55.20%	54.37%	65.84%	47.75%
	F1 score	60.87%	70.48%	56.52%	67.44%	64.58%	66.67%	59.52%	79.61%	55.81%
	Sensitivity	59.57%	78.72%	55.32%	61.70%	65.96%	65.96%	53.19%	87.23%	51.06%
	Specificity	37.04%	22.22%	29.63%	62.96%	33.33%	44.44%	55.56%	44.44%	44.44%
RF	AUC	0.5924	0.5882	0.5610	0.5674	0.5790	0.5898	0.5661	0.6151	0.6123
	Bal. Accuracy	54.41%	50.16%	47.52%	47.24%	48.82%	58.63%	53.86%	53.07%	48.58%
	F1 score	67.37%	61.54%	61.70%	59.34%	57.47%	65.91%	62.92%	63.74%	63.16%
	Sensitivity	68.09%	59.57%	61.70%	57.45%	53.19%	61.70%	59.57%	61.70%	63.83%
	Specificity	40.74%	40.74%	33.33%	37.04%	44.44%	55.56%	48.15%	44.44%	33.33%
XGBoost	AUC	0.5979	0.5992	0.5545	0.5724	0.5601	0.5994	0.5780	**0.6214**	0.6213
	Bal. Accuracy	61.07%	**66.63%**	43.26%	60.99%	50.71%	64.22%	52.01%	52.29%	53.35%
	F1 score	76.92%	**79.21%**	55.56%	63.41%	65.98%	75.00%	62.22%	64.52%	65.96%
	Sensitivity	85.11%	85.11%	53.19%	55.32%	68.09%	76.60%	59.57%	63.83%	65.96%
	Specificity	37.04%	48.15%	33.33%	66.67%	33.33%	51.85%	44.44%	40.74%	40.74%
MLP	AUC	0.5566	0.5662	0.5101	0.5035	0.4922	0.5373	0.5324	0.5684	0.5546
	Bal. Accuracy	59.97%	50.16%	44.84%	59.38%	39.56%	59.42%	49.88%	53.07%	59.42%
	F1 score	69.57%	61.54%	53.49%	56.00%	54.35%	65.12%	59.09%	63.74%	65.12%
	Sensitivity	68.09%	59.57%	48.94%	44.68%	53.19%	59.57%	55.32%	61.70%	59.57%
	Specificity	51.85%	40.74%	40.74%	74.07%	25.93%	59.26%	44.44%	44.44%	59.26%

Table S5. Average performance of classifiers in the interictal state using entropy features with optimal hyperparameter settings.

Classifier	Metric	SampEn	FuzzyEn	SpecEn	RenEn	PermEn	WaveEn	PhasEn	CondEn	DispEn
kNN	AUC	0.5355	0.5179	0.5164	0.5250	0.5296	0.5246	0.5085	0.5067	0.5171
	Bal. Accuracy	46.89%	57.25%	41.06%	45.86%	55.12%	46.14%	51.97%	41.10%	44.29%
	F1 score	33.33%	52.05%	27.27%	44.74%	47.89%	48.10%	53.16%	41.56%	47.50%
	Sensitivity	23.40%	40.43%	19.15%	36.17%	36.17%	40.43%	44.68%	34.04%	40.43%
	Specificity	70.37%	74.07%	62.96%	55.56%	74.07%	51.85%	59.26%	48.15%	48.15%
NB	AUC	0.5310	0.5172	0.5540	0.5438	0.5086	0.5458	0.5069	0.5069	0.5191
	Bal. Accuracy	50.08%	40.03%	52.48%	51.38%	49.49%	55.67%	45.47%	44.80%	52.21%
	F1 score	40.58%	39.47%	48.65%	32.26%	11.54%	54.55%	**71.93%**	42.67%	45.07%
	Sensitivity	29.79%	31.91%	38.30%	21.28%	6.38%	44.68%	**87.23%**	34.04%	34.04%
	Specificity	70.37%	48.15%	66.67%	81.48%	**92.59%**	66.67%	3.70%	55.56%	70.37%
LDA	AUC	0.5075	0.5013	0.5216	0.5407	**0.6012**	0.5127	0.4886	0.5061	0.5171
	Bal. Accuracy	46.69%	41.65%	49.84%	56.97%	59.42%	55.67%	56.23%	46.69%	38.73%
	F1 score	54.12%	48.19%	49.35%	48.57%	65.12%	54.55%	60.24%	54.12%	45.78%
	Sensitivity	48.94%	42.55%	40.43%	36.17%	59.57%	44.68%	53.19%	48.94%	40.43%
	Specificity	44.44%	40.74%	59.26%	77.78%	59.26%	66.67%	59.26%	44.44%	37.04%
LR	AUC	0.5093	0.5012	0.5227	0.5445	0.5971	0.5107	0.4895	0.5084	0.5202
	Bal. Accuracy	46.69%	43.77%	47.71%	54.33%	60.72%	56.74%	59.14%	47.75%	43.50%
	F1 score	54.12%	51.76%	45.33%	49.32%	60.76%	56.41%	62.65%	55.81%	48.78%
	Sensitivity	48.94%	46.81%	36.17%	38.30%	51.06%	46.81%	55.32%	51.06%	42.55%
	Specificity	44.44%	40.74%	59.26%	70.37%	70.37%	66.67%	62.96%	44.44%	44.44%
SVM	AUC	0.4806	0.4855	0.4759	0.5414	0.4979	0.5541	0.4844	0.4944	0.4907
	Bal. Accuracy	55.36%	49.57%	45.78%	40.82%	**61.51%**	47.16%	44.01%	57.76%	54.85%
	F1 score	38.10%	45.95%	11.11%	37.84%	59.74%	37.68%	44.16%	47.06%	44.12%
	Sensitivity	25.53%	36.17%	6.38%	29.79%	48.94%	27.66%	36.17%	34.04%	31.91%
	Specificity	85.19%	62.96%	85.19%	51.85%	74.07%	66.67%	51.85%	81.48%	77.78%
RF	AUC	0.5504	0.5500	0.5521	0.5580	0.5573	0.5346	0.5275	0.5505	0.5431
	Bal. Accuracy	61.23%	47.71%	47.95%	53.27%	59.38%	52.21%	50.91%	51.69%	54.61%
	F1 score	56.76%	45.33%	35.82%	47.22%	56.00%	45.07%	51.28%	50.00%	52.63%
	Sensitivity	44.68%	36.17%	25.53%	36.17%	44.68%	34.04%	42.55%	40.43%	42.55%
	Specificity	77.78%	59.26%	70.37%	70.37%	74.07%	70.37%	59.26%	62.96%	66.67%
XGBoost	AUC	0.5670	0.5495	0.5666	0.5387	0.5577	0.5395	0.5007	0.5493	0.5137
	Bal. Accuracy	54.61%	55.12%	54.06%	52.99%	57.01%	50.87%	56.00%	51.69%	50.08%
	F1 score	52.63%	47.89%	45.71%	43.48%	59.26%	38.81%	52.75%	50.00%	40.58%
	Sensitivity	42.55%	36.17%	34.04%	31.91%	51.06%	27.66%	42.05%	40.43%	29.79%
	Specificity	66.67%	74.07%	74.07%	74.07%	62.96%	74.07%	69.91%	62.96%	70.37%
MLP	AUC	0.4898	0.4781	0.5242	0.4925	0.4827	0.4990	0.4846	0.4916	0.4954
	Bal. Accuracy	37.39%	51.69%	49.05%	55.16%	41.88%	38.69%	49.29%	53.03%	52.76%
	F1 score	40.51%	50.00%	50.63%	58.54%	40.00%	33.33%	42.25%	55.00%	51.95%
	Sensitivity	34.04%	40.43%	42.55%	51.06%	31.91%	25.53%	31.91%	46.81%	42.55%
	Specificity	40.74%	62.96%	55.56%	59.26%	51.85%	51.85%	66.67%	59.26%	62.96%

Table S6. Average performance of classifiers in the dynamic state using entropy features with optimal hyperparameter settings.

Classifier	Metric	SampEn	FuzzyEn	SpecEn	RenEn	PermEn	WaveEn	PhasEn	CondEn	DispEn
kNN	AUC	0.5604	0.5475	0.5796	0.5247	0.5767	0.5777	0.5596	0.5679	0.5767
	Bal. Accuracy	41.10%	58.27%	59.93%	50.32%	53.55%	59.34%	48.78%	58.35%	48.23%
	F1 score	41.56%	41.27%	61.73%	29.51%	50.67%	43.75%	47.37%	63.53%	40.00%
	Sensitivity	34.04%	27.66%	53.19%	19.15%	40.43%	29.79%	38.30%	57.45%	29.79%
	Specificity	48.15%	**88.89%**	66.67%	81.48%	66.67%	**88.89%**	59.26%	59.26%	66.67%
NB	AUC	0.6095	0.6133	0.5870	0.5740	0.5690	0.6023	0.5739	0.6133	0.6104
	Bal. Accuracy	68.44%	68.16%	63.63%	48.27%	53.74%	63.91%	59.42%	65.52%	59.42%
	F1 score	74.16%	72.09%	63.29%	51.85%	25.00%	65.85%	65.12%	71.91%	65.12%
	Sensitivity	70.21%	65.96%	53.19%	44.68%	14.89%	57.45%	59.57%	68.09%	59.57%
	Specificity	66.67%	70.37%	74.07%	51.85%	92.59%	70.37%	59.26%	62.96%	59.26%
LDA	AUC	0.5676	0.5734	0.5507	0.5821	0.5162	0.5692	0.5617	0.5781	0.5699
	Bal. Accuracy	70.57%	58.87%	57.25%	47.20%	58.08%	57.80%	50.91%	56.50%	63.40%
	F1 score	76.92%	60.00%	52.05%	50.00%	60.98%	58.23%	51.28%	62.79%	68.97%
	Sensitivity	**74.47%**	51.06%	40.43%	42.55%	53.19%	48.94%	42.55%	57.45%	63.83%
	Specificity	66.67%	66.67%	74.07%	51.85%	62.96%	66.67%	59.26%	55.56%	62.96%
LR	AUC	0.6031	0.6066	0.5658	0.5942	0.5889	0.5802	0.5963	0.6073	0.6009
	Bal. Accuracy	65.52%	64.46%	57.29%	53.03%	63.91%	63.91%	47.20%	60.48%	62.33%
	F1 score	71.91%	70.45%	61.90%	55.00%	65.85%	65.85%	50.00%	66.67%	67.44%
	Sensitivity	68.09%	65.96%	55.32%	46.81%	57.45%	57.45%	42.55%	61.70%	61.70%
	Specificity	62.96%	62.96%	59.26%	59.26%	70.37%	70.37%	51.85%	59.26%	62.96%
SVM	AUC	0.5976	0.5963	0.5608	0.5224	0.5764	**0.6279**	0.5882	0.5346	0.5286
	Bal. Accuracy	69.50%	**72.42%**	63.63%	65.76%	62.88%	70.80%	52.25%	55.67%	55.40%
	F1 score	75.56%	**77.78%**	63.29%	66.67%	71.74%	72.29%	56.10%	54.55%	51.35%
	Sensitivity	72.34%	**74.47%**	53.19%	57.45%	70.21%	63.83%	48.94%	44.68%	40.43%
	Specificity	66.67%	70.37%	74.07%	74.07%	55.56%	77.78%	55.56%	66.67%	70.37%
RF	AUC	0.5947	0.5837	0.5920	0.5598	0.5883	0.6218	0.5859	0.5954	0.5877
	Bal. Accuracy	64.97%	57.01%	64.97%	50.63%	59.69%	66.82%	68.95%	64.97%	63.91%
	F1 score	67.47%	59.26%	67.47%	48.00%	67.42%	68.29%	71.43%	67.47%	65.85%
	Sensitivity	59.57%	51.06%	59.57%	38.30%	63.83%	59.57%	63.83%	59.57%	57.45%
	Specificity	70.37%	62.96%	70.37%	62.96%	55.56%	74.07%	74.07%	70.37%	70.37%
XGBoost	AUC	0.6042	0.5934	0.5853	0.5639	0.5961	0.6191	0.5967	0.5913	0.5902
	Bal. Accuracy	67.89%	56.23%	68.12%	56.19%	56.78%	70.02%	62.88%	52.52%	61.27%
	F1 score	69.88%	60.24%	64.00%	50.00%	65.17%	72.94%	71.74%	58.82%	65.88%
	Sensitivity	61.70%	53.19%	51.06%	38.30%	61.70%	65.96%	70.21%	53.19%	59.57%
	Specificity	74.07%	59.26%	85.19%	74.07%	51.85%	74.07%	55.56%	51.85%	62.96%
MLP	AUC	0.5710	0.5700	0.5388	0.5271	0.5599	0.5392	0.4854	0.5774	0.5685
	Bal. Accuracy	70.57%	45.90%	58.59%	49.57%	59.42%	67.34%	52.76%	54.37%	57.29%
	F1 score	76.92%	55.17%	57.14%	45.95%	65.12%	64.94%	51.95%	59.52%	61.90%
	Sensitivity	**74.47%**	51.06%	46.81%	36.17%	59.57%	53.19%	42.55%	53.19%	55.32%
	Specificity	66.67%	40.74%	70.37%	62.96%	59.26%	81.48%	62.96%	55.56%	59.26%
